# New bis-isoxazole with monoterpenic skeleton: regioselective synthesis, spectroscopic investigation, electrochemical, and density functional theory (DFT) studies

**DOI:** 10.3906/kim-2109-28

**Published:** 2021-12-18

**Authors:** Ali OUBELLA, Meryem HRIMLA, Mouhi Eddine HACHIM, Mourad FAWZI, Abdoullah BIMOUSSA, Lahoucine BAHSIS, Aziz BOUTOUIL, Aziz AUHMANI, Abdelkhalek RIAHI, My Youssef AIT ITTO

**Affiliations:** 1 Laboratory of Organic Synthesis and Physico-Molecular Chemistry, Department of Chemistry, Faculty of Sciences Semlalia, Marrakech, Morocco; 2 Laboratory of Analytical and Molecular Chemistry, Polydisciplinary Faculty, Cadi Ayyad University, Safi, Morocco; 3 Laboratory of Coordination and Analytical Chemistry (LCCA), Department of Chemistry, Faculty of Sciences of El Jadida, Chouaïb Doukkali University, El Jadida, Morocco; 4 MSO team, CNRS UMR 7312 Institute of Molecular Chemistry University of Reims Champagne-Ardenne, Bat. Europol’Agro - Moulin de La Housse UFR Sciences, Cedex 2, France

**Keywords:** Bis-isoxazone, [3+2] cycloaddition reaction, regioselectivity, density functional theory (DFT) calculations, cyclic voltammetry

## Abstract

A novel bis-isoxazole was synthesized from (R)-Carvone and p-methylbenzaldoxime, via two successive [3+2] cycloaddition reactions (**32CA**). The newly obtained bis-isoxazole has been fully characterized by HRMS and NMR spectroscopy. The HMBC experiment was performed to determine the stereo and the regioselectivity of the reaction. The electrochemical behavior of the studied compound, in oxidation and reduction processes, was examined using the cyclic voltammetry technique. In addition, the regioselectivity of the [3+2] cycloaddition reaction and the molecular structure of the title compound was performed by density functional theory (DFT). The HOMO and LUMO orbitals were investigated to determine the electronic properties of the synthesized compound. Besides, the global reactivity indexes were used to explain the regioselectivity for the formation of the bis-isoxazole, the theoretical results are in good agreement with experimental findings.

## Graphical Abstract

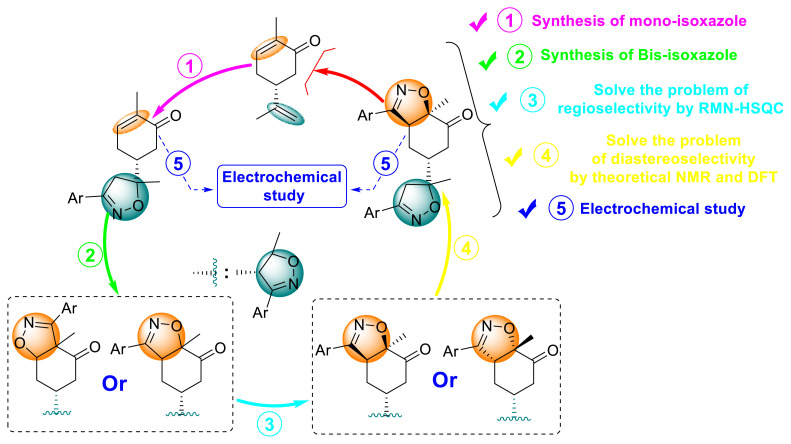

## 1. Introduction

Because of their heteroatomic constitution and their spatial disposition, the heterocycles have attracted much attention in innumerable fields of application [1–3], including isoxazoles especially in pharmacology, as they show various biological activities, including antioxidant [4], nematicidal [5], and antiviral [6]. Recently, isoxazoles have been widely studied as corrosion inhibitors and have revealed remarkable inhibition efficacy [7].

Among several isoxazole synthesis procedures, the [3+2] cycloaddition reactions of nitrile oxides with alkynes or alkenes can be ısed in various ways because of their straightforwardness and their efficiency [8–10]. While transition metal-mediated preparations of 3,4-disubstituted and 3,4,5-trisubstituted isoxazoles have been reported to provide remarkable yields [11–12], other efficient procedures use iodine compounds such as PhI(OCOCF_3_)_2_ [13], t-BuOI [14] or PhI(OCOCH_3_)_2_ [15–16] with oximes. Since oximes can be conveniently generated in considerable yield, the intramolecular cyclization of α,β-unsaturated oximes, or β-keto oximes constitutes the fundamental step for the successful synthesis of the isoxazole nucleus [17–18].

Nowadays, the science of molecular electrochemistry has become a crucial tool of research efforts in order to develop new renewable energy technologies that meet environmental conditions. The urgency of these environmental needs is well represented by the rapid evolution [19–23]. In the synthesis of heterocyclic compounds, organic electrochemistry is, without a doubt, one of the milder and more environmentally benign tools available to chemists [24–27]. In terms of mechanism, electrochemistry is a useful tool to examine reactions involving electron transfers or a structural modification, often resulting in the oxidation or reduction of a metal complex [28]. Among the electrochemical techniques, cyclic voltammetry is the most popular and commonly employed technique to investigate the reduction and oxidation processes of molecular species.

**Figure 1:**
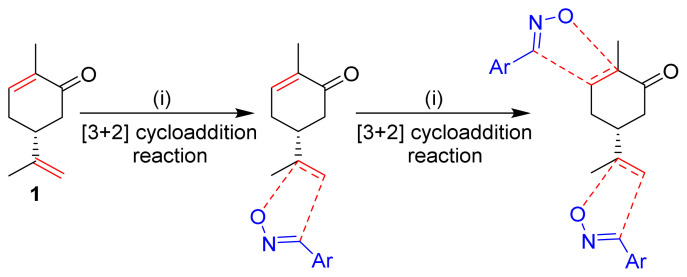
The design strategy of the new bis-isoxazole hybrid.

These bibliographic findings prompt us to expand our research work aiming at the preparation and theoretical study of heterocyclic systems with monoterpenic skeleton [29–32]. The target compound was designed by the incorporation of two isoxazole coresintoa monoterpenic skeleton by reacting (R)-carvone with an arylonitrile oxide via two sequential 1,3-dipolar cycloaddition reactions (Figure 1). Through the obtained results, we discussed the electronic and structural effects in order to carry out a comparative study of its electrochemical properties.

We report also an experimental NMR investigation of the formation of the uniquely obtained bis-isoxazole regioisomer **3**, followed by a study on the plausible mechanism for this regioselective synthesis of the stoichiometric compound **3** via the local reactivity indexes. HOMO, LUMO, and NBO analyses were performed using density-functional theory (DFT). The electrochemical activity of the studied compounds (**2** and **3)** was carried out using the cyclic voltammetry technique.

## 2. Experimental section

### 2.1. Instruments and reagents

All chemicals were used as obtained from commercial sources (Aldrich and Acros). Melting points (m.p.) were determined using a capillary apparatus and are inaccurate. Analytical thin-layer chromatography (TLC) was performed on plates precoated with E. Merck silica gel 60 F254 to a thickness of 0.25 mm. HRMS were obtained on a Q-TOF micromass spectrometer. ^1^H and ^13^C NMR spectra were recorded in CDCl_3_ with a 500 MHz Bruker Avance III spectrometer with a BBFO + probe. Chemical shifts (δ) are expressed in parts per million (ppm). They were recorded relative to solvent CDCl_3_ signal (7.26 ppm and 77.16 ppm). The (R)-carvone-isoxazole **2** was prepared according to the reported method [33].

### 2.2. Cyclic voltammetry

All the measurements were carried out using a potentiostat equipped with a three-electrode cell, consisting of two Pt electrodes (diameter = 0.1 cm) as working and counter-electrode as well as a saturated calomel reference electrode (SCE) (3 M KCl), all connected to a Voltalab 10 system (PGZ 100 radiometer) controlled by the Volta master 4 software. The potential was scanned between −2000mV and +2000mV at a scan rate ranging from 50 to 1000 mV/S. As the electrolyte, an anhydrous acetonitrile (CH_3_CN) solution containing the synthesized compound (**2** or **3**) and tetrabutylammonium perchlorate (Et_4_ClNO_4_) in concentrations of 10-3 and 0.1 M, respectively was used at room temperature. UV-Vis spectra were recorded using a UV-6300 PC double-beam spectrophotometer (200–800 nm) operating at 1 nm resolution with a scanning speed of 100 nm min-1 at room temperature. All the measurements were carried out in acetonitrile containing a concentration of 10^−6^ M for two compounds studied.

### 2.3. Synthesis

To a stirred solution of mono-isoxazole **2** (1eq: 6.57 mmol) and p-methyl benzaldoxime (0.5eq, 1eq: 6.57 mmol, 1.5eq, 2eq, 2.5 eq, 3eq, 3.5eq and 4eq) in CH_2_Cl_2_ (30 mL), aqueous NaOCl was added dropwise (during 30 min) at 0 °C (5.2%; 15.65 mmol for 1 equivalent) according to Table 1.

After stirring for 10 min (the reaction was monitored by TLC) at room temperature, the layers were separated, and the aqueous layer was extracted with CH_2_Cl_2_ (3 × 10 mL). The combined organic extracts were dried over anhydrous Na_2_SO_4_ and the solvent was evaporated in vacuo. The residue was purified by column chromatography using hexane/Ethylacetate mixture (82:18) as eluent. The structures, spectroscopic analysis (HRMS), and the systematic name (according to IUPAC) of two products **2** and **3** are mentioned in Table 2.

#### (3aS,5R,7aR)-7a-methyl-5-(5-methyl-3-(p-tolyl)-4,5-dihydroisoxazol-5-yl)-3-(p-tolyl)-3a,4,5,6-tetrahydrobenzo[d]isoxazol-7(7aH)-one (3)

Yield 11%; white solid: mp = 112±2 °C (Ethanol); HRMS (TOF-MS ES+) (m/z): found 417.2127 [M+H]^+^, calculated 417.2128. ^1^ H NMR δ (ppm): 1.34 (3H, s); 1.42 (3H, s); 1.75 (2H, m); 1.98 (2H, m); 2.33 (3H, s); 2.28 (3H, s); 2.74–2.76 (1H, m); 2.77–3.10 (2H, m); 3.75 (1H, dd *J* = 8.75 & 1.45 Hz); 7.00–7.80 (8H, m). ^13^C NMR δ (ppm): 20.06 (CH_3_); 21.43 × 2 (2 × CH_3_); 24.37 (CH_3_); 26.40 (CH_2_); 39.60 (CH_2_); 40.09 (CH); 44.69 (CH_2_); 55.60 (CH); 87.69 (C5′); 87.79 (C7a); 124.84 (CAr); 126.54 (HCAr); 126.76 (CAr); 127.76 (HCAr); 129.49 (HCAr); 129.91 (HCAr); 140.14 (CAr); 141.16 (CAr); 155.86 (C3′=N); 158.66 (C3=N); 205.83 (C=O).

### 2.4. Computational methods

The quantum chemical calculations were performed using GAUSSIAN 09W [34] through the DFT/B3LYP method and 6-311G(d,p) basis sets. All the frequencies obtained are positive, which proves that the structure corresponds to minimum energy [35–38]. The electronic chemical potential (μ) and chemical hardness (η) are calculated using energies of the frontier molecular orbital HOMO (E_H_) and LUMO (E_L_) as follows μ= (E_H_ + E_L_)/2 and η= (E_L_ - E_H_). The global electrophilicity (ω) and nucleophilicity (*N*) indexes were measured at the same level and are given by the following simple expressions: ω= μ^2^ / 2η; N = E_H_ − E_H_ (tetracyanoethylene (TCE)) [39–44]. The Parr functions are calculated using the Mulliken atomic spin densities [45].

## 3. Results and discussion

### 3.1. Chemistry

**Scheme 1:**
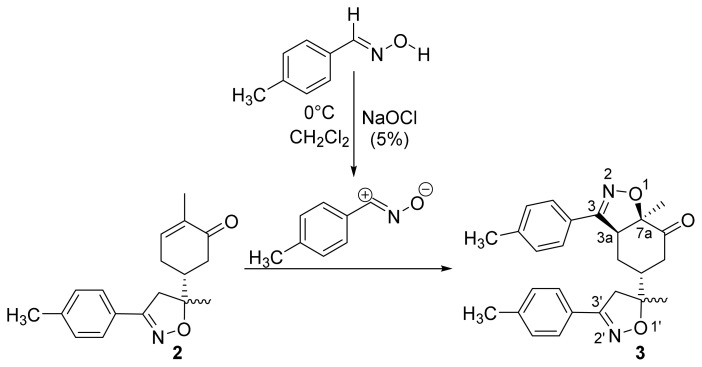
Synthetic route to bis-isoxazole hybrid compound **3**.

The first step in the synthesis of the desired hybrid bis-isoxazole **3** from (R)-carvone is the preparation of mono-adduct **2** according to the procedure reported in our previous work [33]. The second step was achieved through [3+2] cycloaddition reaction by treating the mono-adduct **2**, with a stoichiometric amount of *p*-methyl arylonitrile (generated in situ from the corresponding oxime using 5% bleach solution). The reaction was performed at 0 °C, in dichloromethane as a solvent to yield the corresponding bis-isoxazole **3** in a high stereo and regioselective manner with low yield (11%) (Scheme 1).

The low yield (11%) of the bis-isoxazole **3** has prompted us to carry out an optimization study of the reaction yield by increasing the number of equivalents of *p*-methylbenzonitrile oxide. The obtained results are shown in Table 3.

As revealed in Table 3, the yield of the reaction increases with the increase of the number of equivalents of p-methyl arylonitrile, such as the use of three equivalents of dipole gave the desired compound with an improved yield of 48 %. On the other hand, under the same operating conditions, there is a drop in yield from 48% to 41% in the case of 3.5 and 4 equivalents. This drop-in yield is likely due to the effect of the excess dipole on the formed bis-isoxazole **3**.

#### ^1^H NMR and ^13^C NMR spectrum analysis of compound 3

The mono-adduct **2** was synthesized and identified according to the reported work [33]. Similarly, the newly synthesized bis-isoxazole **3** was fully characterized from its spectral NMR (1D & 2D) and HRMS data. In HRMS spectrometry, bis-isoxazole **3** reveals its pseudo molecular ion at m/z = 417.2127 [M+H]^+^, which is consistent with the molecular formula C_26_H_28_N_2_O_3_. In NMR spectra of the hybrid compound **3**, and in comparison with those of starting mono-adduct **2**, we can easily state that the attack of the 1,3-dipole occurs on the internal double bond of the terpenic skeleton. Indeed, the first noteworthy data ARE the disappearance of resonances (δ ^1^H 6.76 ppm; δ ^13^C 144.50 ppm), characterizing the methane group (HC=) of the internal double bond of the precursor **2**. These letters are replaced in δ ^1^H NMR spectra by one hydrogen doublet of doublet δ ^1^H 3.75 ppm *J* = 1.1 & 6.8 Hz attributed to the proton at C3a position, while in ^13^C NMR spectra, we noted two shielded signal at 55.60 and 87.79 ppm assigned respectively to C3a and C7a of isoxazole.

**Figure 2:**
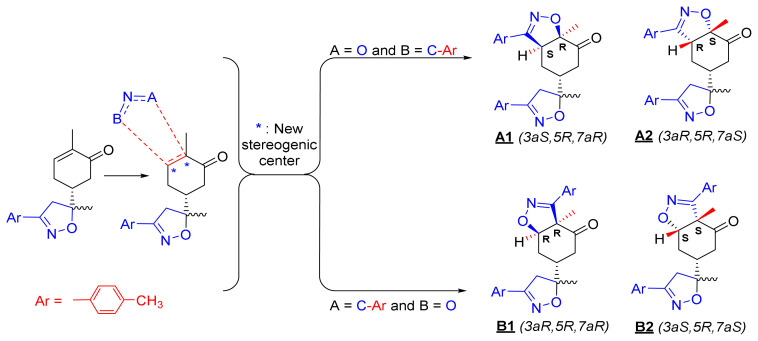
Selected reaction paths associated with the [3+2] cycloaddition reaction of *p*-methyl benzonitrileoxid with (R)-carvone-isoxazole **2**.

The 1,3-dipolar cycloaddition reaction of the 4-methylbenzonitrile oxide on the C=C dipolarophile of compound **2** can theoretically take place at both or one of its two faces according to two opposite directions leading to the formation of two possible regioisomers (**A** & **B**) each in the form of two possible diastereomers (**A**: **A1** and/or **A2**) and (**B**: **B1** and/ or **B2**) (Figure 2).

**Figure 3:**
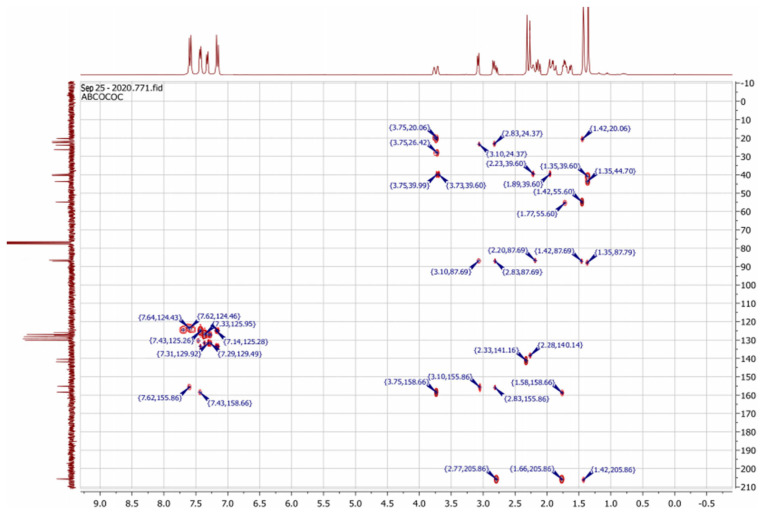
The HMBC correlations of bis-isoxazole-carvone **3**.

To resolve this structural problem, 2D (two-dimensional) NMR spectroscopy was carried out, and HMBC (heteronuclear multiple bond correlation) experiments (Figure 3) allowed us to rule out **B1** and **B2** and confirm the **A1** or **A2** stereoisomers.

**Figure 4:**
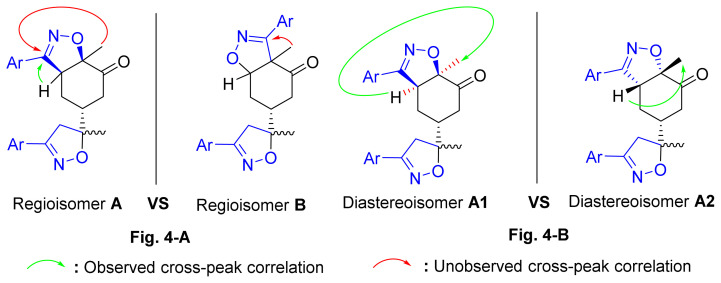
**A, B**. Main HMBC cross-peaks correlations were observed in the 2D-NMR spectra of compound **3**.

Indeed, in the HMBC spectrum (Figure 3), we note a ^2^*J*_CH_ correlation between the C3a-H proton (δ ^1^H 3.75) and the C3=N imine carbon (δ ^13^C 158.66) of the isoxazole ring, while no correlation was observed between this latter and the protons of C7a-CH_3_ methyl group (δ ^1^H 1.42). This deep examination of the HMBC spectrum informs us about the regioselective formation of isomer **A** (**A1** or **A2**) (Figure 4-A). In the same spectrum, we can also note a ^3^*J*_CH_ correlation between the C3a-H proton (δ ^1^H 3.75) and the methyl carbon at the C7a position. This three bond correlation would be present in both **A1** and **A2** diastereoisomers (Figure 4-B).

**Figure 5:**
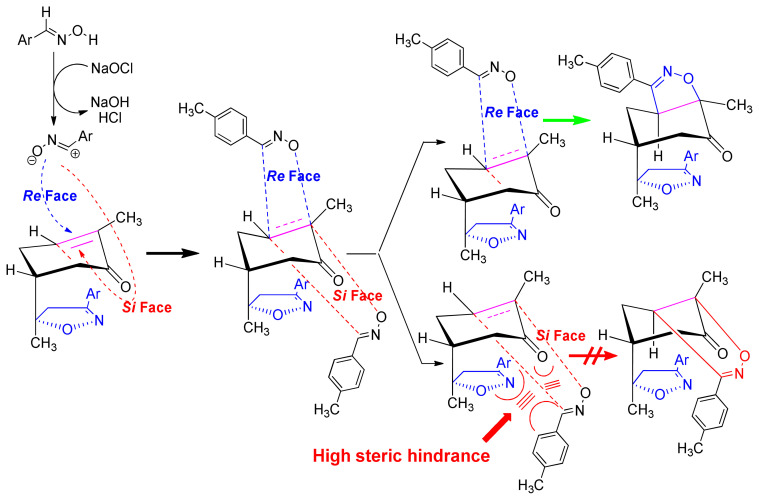
The proposed mechanism of the formation of the stereoisomer A1 of bis-isoxazoline 3.

Thus, the 1,3-dipolar cycloaddition reaction of 4-methylbenzonitrile oxide on isoxazoline **2** was highly regioselective. This result corroborates the one obtained in our recently published study, which showed that the C3 carbon (in beta position of the carbonyl group) is more nucleophilic than the C2 carbon (in alpha position of the carbonyl group) [45]. Therefore, the bis-isoxazoline **3** is obtained in a unique manner where the oxygen atom of the 1,3-dipole is linked to the more hindered carbon of the cyclohexenic double bond of the isoxazoline **2**. Furthermore, the 1,3-dipolar cycloaddition reaction of 4-methylbenzonitrile oxide on isoxazoline **2** was revealed to be also highly diastereoselective. This could be ascribed to the fact that the *Si* face of the cyclohexenic double bond of **2** is much more sterically hindered than the *Re* face. Therefore, the approach of the 1,3-dipole on the isoxazoline **2** should be favoured on the *Re* face thus producing the obtained **A1** diastereoisomer (Figure 5).

If the regioselectivity of the reaction was unambiguously established, its diastereoselectivity and, so, the absolute configuration of the two newly formed asymmetric carbons C3a and C7a remained unknown. In an attempt to resolve this problem, we have carried out a DFT. Theoretical study where ^1^H and ^13^C NMR chemical shifts of compound **3** were calculated at the B3LYP/6-31G(d,p) using GIAO approach in the solvent phase. The calculations were performed in CDCl_3_ solvent by using C-PCM formalism, and the selected shifts were compared with the experimental values as illustrated in Table 4.

According to Table 4, we can see that the highest chemical shift value was that of the carbonyl group (C7 atom), which was observed experimentally at 205.83 ppm, while the corresponding calculated values were 214.64 ppm for **A1** and 219.97 ppm for **A2**. Concerning the C8 and C9 carbon atoms of the two methyl groups, which were revealed with the lowest chemical shift values, they were observed experimentally at 20.06 and 24.37 ppm, while, theoretically, they were noted at 19.52 and 25.27 ppm for diastereomer **A1** and 17.61 and 23.01 ppm for diastereomer **A2**. A slight difference was observed between the C5′ and C7a isoxazolic carbons; their experimental chemical shift values were recorded respectively at 87.69 and 87.79 ppm, while their theoretical ones were recorded at 94.51 and 92.30 ppm for **A1**, and at 95.96 and 96.33 ppm for **A2**, respectively.

Among the two Csp^3^ methine groups, the one at C3a position (^1^H 3.76, ^13^C 55.79) is more deshielded than the one at C5 position (^1^H 2.35, ^13^C 39.10) because of the inductive withdrawing effect of the adjacent imine group. The calculated chemical shift values of C3a-H methine group were (^1^H 3.59, ^13^C 59.71) for **A1** and (^1^H 3.41, ^13^C 61.66) for **A2**. On the other hand, those of C5-H methine group were (^1^H 2.24, ^13^C 39.53) for the diastereomer **A1** and (^1^H 1.99, ^13^C 43.41) for the diastereomer **A2**.

**Figure 6:**
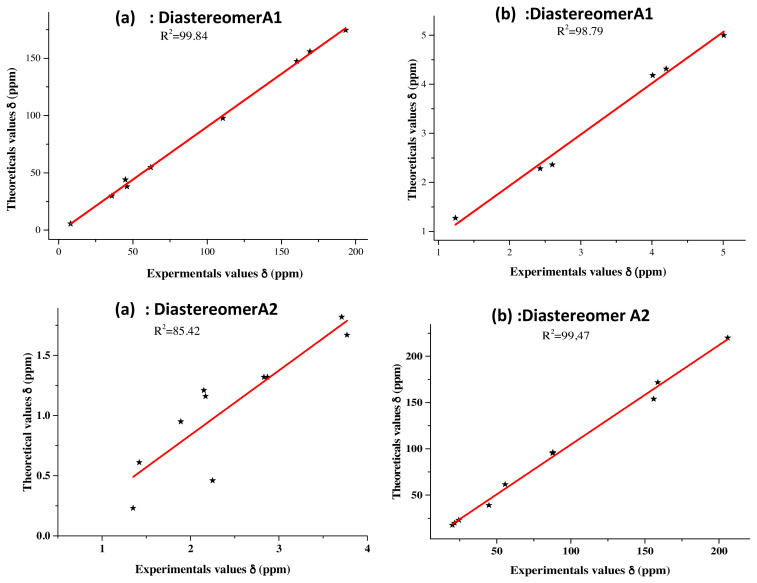
Correlation curves between the predicted and experimental ^1^H NMR. (**a**) and ^13^C NMR, (**b**) chemical shifts for product **3**.

The conclusion to be drawn concerning the comparability between the experimental and the theoretical findings is that the experimental NMR data are consistent with the computed values from the optimized structure of diastereomer **A1** (Figure 6).

According to this NMR analysis, which allowed an approximate assignment of each carbon and its protons, we can relatively conclude that the absolute configuration of both new stereogenic centers (C3a & C7a) is 3aS and 7aR.

### 3.2. IR spectral analysis

Subsequently, an FT-IR spectral study of the title compound was carried out. The structure of compound **3** was confirmed according to its IR spectral data, the existence of the band at υ_max_ = 1720 cm^−1^ corresponds to the carbonyl group (C=O). The characteristic band of the aromatic nucleus (C=C) has been revealed at 1515 cm^−1^. However, the scaled harmonic vibrational frequencies of compound **3** were calculated from the optimized structure with DFT/B3LYP using a 6–31G(d,p) basis set at room temperature in the region between 400 and 4000 cm^−1^. The experimental and theoretical spectrums plotted on the transmittance (%) against the wavenumber (cm^−1^) are shown in Figure 6a,6b.

The calculated frequencies of the diastereoisomer **A1** and **A2** were compared with the observed values, and the results found are grouped in Table 5.

### 3.3. Mechanistic study

**Figure 7:**
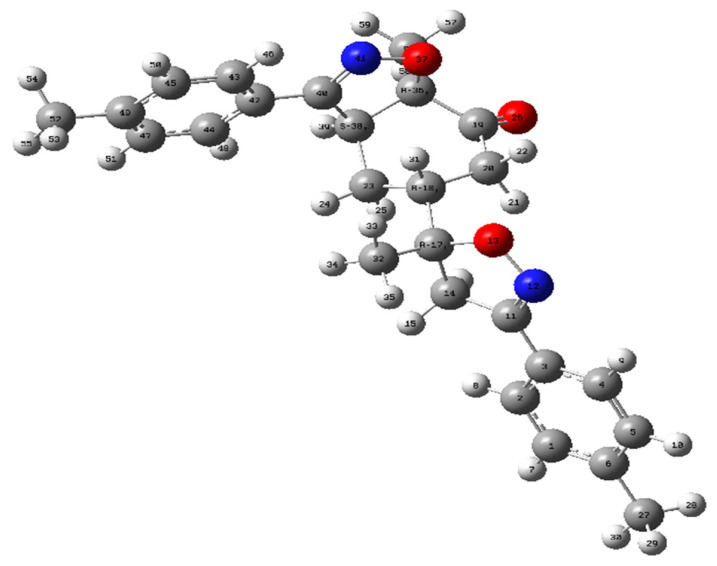
Optimized geometry of product **3** atB3LYP/6-311++G(d,p) level.

The geometric structure of compound bis-isoxazole hybrid **3** (**A1**) was optimized using DFT at B3LYP/6-311G(d,p) level (Figure 7).

**Figure 8:**
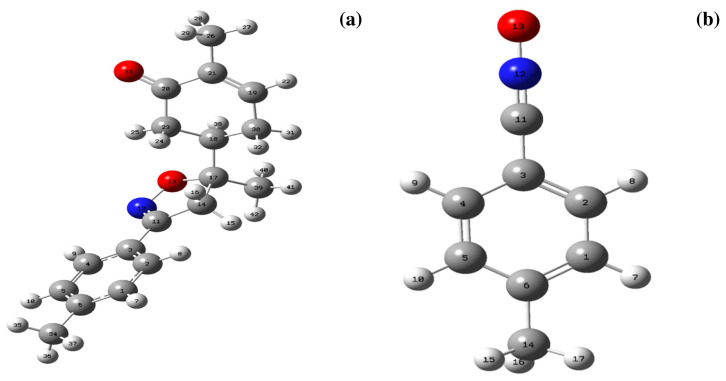
Optimized structures of the **2(a)** and dipole**(b)** at B3LYP/6-311G(d,p) level.

**Scheme 2:**
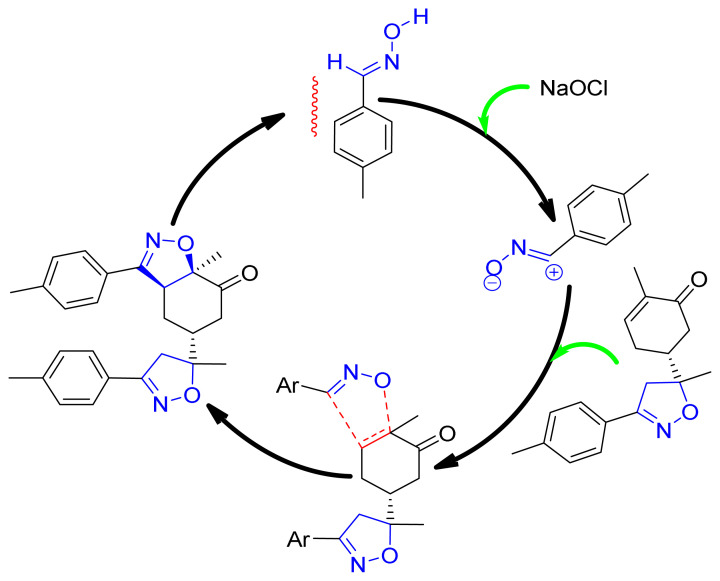
The cycloaddition reaction between **2** and dipole.

The cycloaddition reaction of compound **2** in the presence of the 1,3-dipole is illustrated in Scheme 2. To get a closer understanding of the regioselectivity experimentally, DFT calculations were performed at the B3LYP/6-311G(d,p) level of both reagents. The optimized geometries obtained and the numbering of the atoms of the most stable conformation of both reactants are shown in Figure 8.

The global reactivity indexes are potent tools that can explain the reaction mechanism and are measured through the global electron density transfer (GEDT) value [46–47]. In this regard, the global electrophilicity and nucleophilicity indexes are calculated and summarized in Table 6. The electronic chemical potential value of dipole, **μ** = −3.94 eV, is closer to that of the compound **2**, **μ** = −3.88 eV. These results confirm that this 32CA reaction will have a nonpolar character. The electrophilicity ω indices (1.63 and 1.54 eV) and the nucleophilicity N indices (2.93 and 2.65 eV) of the compound **2** and dipole illustrate that both reagents act as moderate electrophiles and moderate nucleophiles within the electrophilicity and nucleophilicity scales, confirming the nonpolar character of this cycloaddition reaction and suggests high activation energy for the formation of the corresponding products [48].

**Figure 9:**
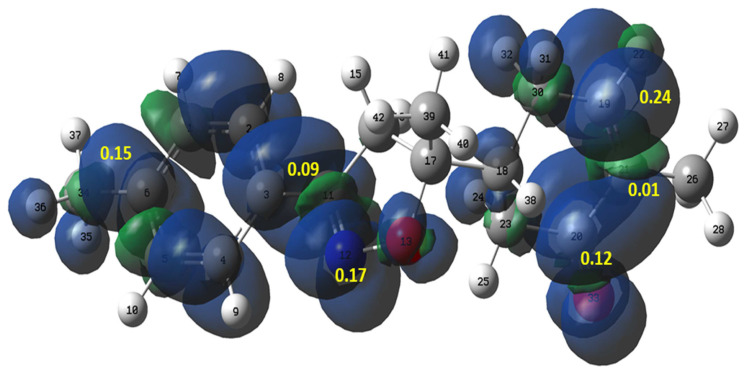
Three-dimensional representation of the Mulliken atomic spin densities of **2** together with the P_k_^+^ Parr functions values.

Recently, Domingo et al. have proposed the Parr functions as a helpful tool to explain the regioselectivity that is experimentally observed in 32CA reactions [49]. The authors confirmed that the ethylene group could participate in 32CA reactions by forming the first new single bond through their most electrophilic center. Therefore, the local reactivity of compound **2** was analyzed by the electrophilic P_k_^+^ Parr functions (Figure 9). The P_k_^+^ Parr function analysis indicates that the carbon C19 atom with a P_k_^+^ value of 0.24 is more electrophilic center than the carbon atoms C20 and C21, with P_k_^+^ values of 0.12 and 0.01, respectively. These results indicate that the first single bond formation will involve the most electrophilic C19 carbon explaining the experimentally observed results.

**Figure 10:**
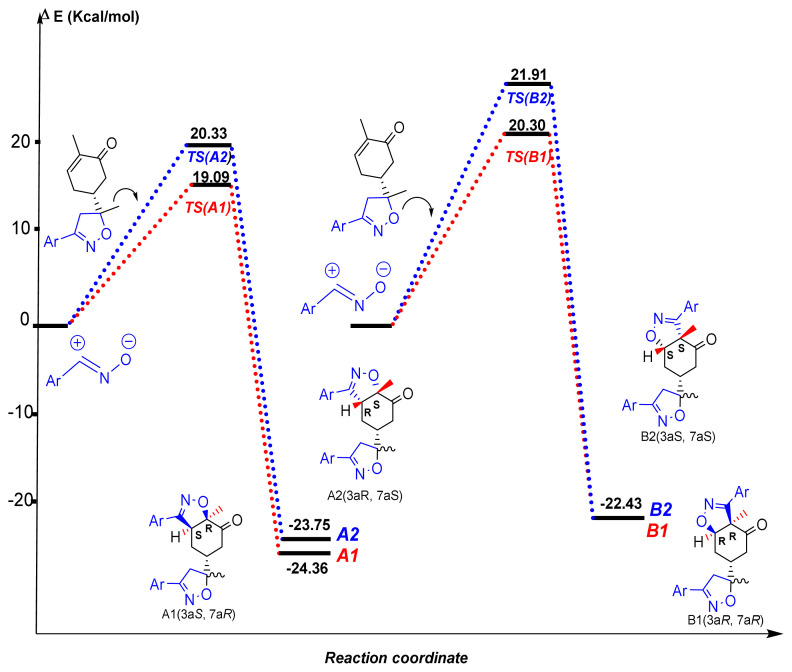
Relative energie for the studied reactions paths of the 32CA of benzonitrile oxide with compound **2**.

In order to illuminate the experimental results, two reaction paths associated with regioisomeric approach modes to the regio- and diastereofacial attacks for this 32CA reaction between product **2** and dipole were considered (Figure 9). The results indicate that the above-mentioned reaction paths proceed via a one-step mechanism, and the relative energies are donated in Figure 10.

The activation energies associated with the reaction paths conduct to the formation of regioisomeric **A** are 19.09 and 20.33 kcal/mol for **A1** and **A2**, respectively. In the case of the regioisomeric **B**, the results were found to be 20.30 and 21.91 kcal/mol for the compounds **B1** and **B2**, respectively. These results confirm that the formation of the compounds **A1** and **A2** are more exothermic than the formation of **B1** and **B2**. Also, the activation energy associated with TS-A1 (1.321 kcal/mol) is lower than that associated with TS-B1. The theoretical results are in good agreement with the experimental observations (Figure 10).

### 3.4. Electrochemistry study

#### 3.4.1. Cyclic voltammetry measurements

**Figure 11:**
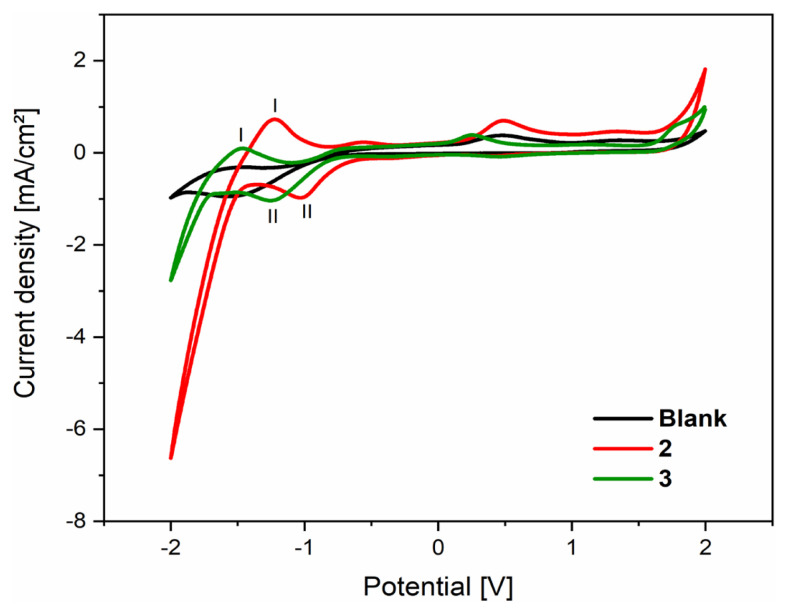
Cyclic voltammograms of (Black) Blank, (Red) 10^−3^M of compound **2**, and (Green) 10^−3^ M of compound **3** in 0.1 M Et_4_NClO_4_/ acetonitrile solution at 200 mV/s at room temperature.

The electrochemical properties of compounds **2** and **3** were performed by cyclic voltammetry in acetonitrile solution at a scan rate of 200 mV/s in the potential range from 2 to −2 V at room temperature are shown in Figure 11, and their electrochemical data are given in Table 5. The analysis of cyclic voltammetry spectra of the **2**, **3**, and their comparison with the blank spectrum show that new anodic and cathodic peaks appear as indicated in Figure 11. Indeed, the anodic current peaks observed around −1.22 and −1.48 V for compounds **2** and **3**, respectively, are located in a negative range and precede the cathodic current peaks, which appear around −1 and −1.25 V for compounds **2** and **3**, respectively; this indicates that it is not a Red/Ox system of the same grouping in the molecules, which can be explained by the irreversibility of the examined compounds [50–51]. On the other hand, these oxidation peaks observed in Figure 11 can be explained by oxidation at the α-position of the carbonyl group or/and the α-position of the double bonds, knowing that the hydrogens at these positions are labile. Then, the reduction peaks may be due to protonation of nitrogen atoms or/and reduction of nonaromatic double bonds or the carbonyl group to alcohol.

**Figure 12:**
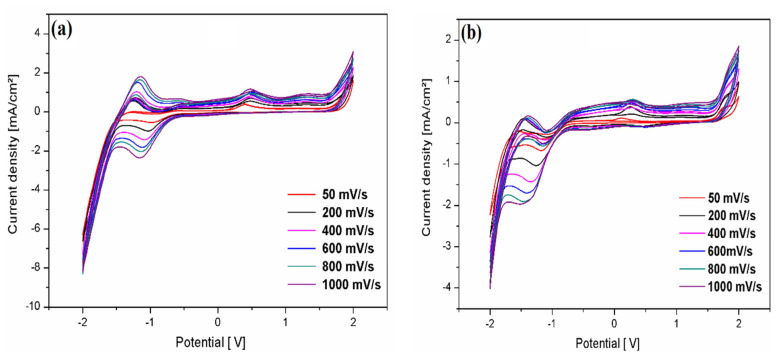
Cyclic voltammograms of 10^−3^ M of **2**, and **3** compounds in a solution of 0.1 M Et_4_NClO_4_/ acetonitrile at different scanning speeds at room temperature.

Figure 12 represents the effect of scan rate on the simulated voltammograms for 10^−3^ M of compounds **2** and **3** in a 0.1 M Et_4_NClO_4_/acetonitrile solution at different scan rates (50–1000 mV/s) at room temperature. The peak current intensities of both compounds increase with increasing scan rate. However, the oxidation and reduction peaks of compound **2** have the same appearance as that of compound **3**, but they move in the negative direction with a higher height of current intensities than compound **2**. This phenomenon can be attributed to the steric effect of the 5-methyl-3-p-tolyl-4,5-dihydroisoxazole group, which influences the electrochemical properties at the electrode surface.

On the other side, the HOMO, LUMO energy levels, and corresponding band gaps E_g_^CV^ of the two compounds were also determined by using the cyclic voltammetry method by measuring the first oxidation ( $E_{ox}^{onset}$) and the first reduction ( $E_{red}^{onset}$) potentials of two studied compounds (as shown in Table 7) according to equations (1) and (2) [52–54]:


(1) $${\text{E}}_{HOMO}=-{\text{E}}_{\text{ox}}^{\text{onset}}-4.8$$

(2) $${\text{E}}_{LUMO}=-{\text{E}}_{\text{red}}^{\text{onset}}-4.8$$

Where ( $E_{ox}^{onset}$) and ( $E_{red}^{onset}$) represent the onset oxidation and reduction potential values relative to the ferrocene/ferricenium couple, respectively with E_ref_ = −4.8 eV.

The band gaps E_g_^CV^were calculated from the difference between the onset of oxidation and reduction, according to the following equation [52–54]:


(3) $${\text{E}}_{\text{g}}^{\text{CV}}={\text{E}}_{\text{ox}}^{\text{onset}}-{\text{E}}_{\text{red}}^{\text{onset}}$$

The band gaps (E_g_^CV^) of compound **3** are higher than those of compound **2**; this can be attributed to the increased size of molecule **3**. Compared to compound **2**, compound **3** has higher stability due to the lower HOMO levels. However, the LUMO energy level of compound **2** is higher than that of compound **3**, which allows for electron transfer after excitation.

#### 3.4.2. Optical properties

**Figure 13:**
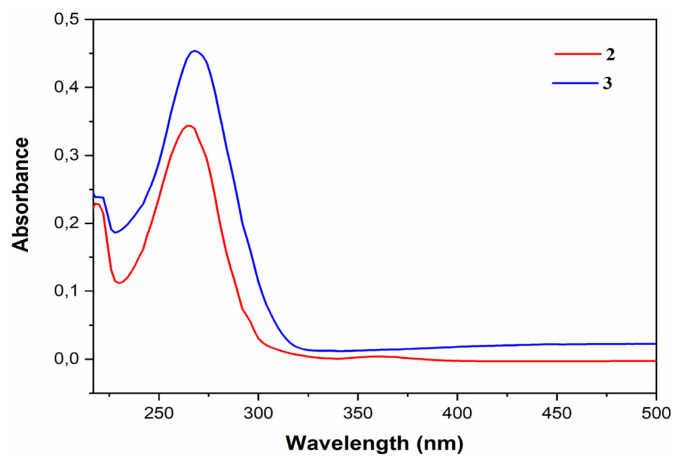
UV-Vis absorption spectra of compounds **2** (red) and **3** (blue) in acetonitrile at room temperature.

The UV/Vis experimental spectra are shown in Figure 13. The maxima for compounds **2** and **3** are located in the UV-Vis at λ_max_ = 218.81; 265.19 nm (Abs = 0.2263 and 0.3427, respectively) and at λ_max_ = 220.09; 268.40 nm (Abs = 0.2402 and 0.4538, respectively), respectively. Comparing these two experimental spectra, we can note that the spectrum of compound **3** shows a slight shift and has a higher absorbance value than compound **2** due to its stronger conjugate effect [55]. This shift corresponds to an optical band gap (E_g_^Opt^) from 5.35 eV (compound **2**) to 5.37 eV (compound **3**) (Table 8). The excellent absorption properties of compound **3** are associated with the large delocalization of the most occupied molecular orbital (HOMO) and the least occupied molecular orbital (LUMO) [56]. These transitions can be measured using the energy of the long-wave edge of the exciton absorption band [57]. The optical band gap (E_g_^opt^) values have been calculated according to equation (4) [58] and are presented in Table 6. However, the difference between E_g_^CV^ and E_g_^Opt^ is due to the progression of reduction, oxidation, and the difference in energy level between the LUMO of the electron transport units and the HOMO of hole transport units, respectively. This result has been confirmed by DFT calculations.


(4) $$E_{g}(ev)=\frac{1242}{\lambda_{\text{onset}}\;(\text{nm})}$$

## 4. Conclusion

In this study, we have described a synthesis of new chiral bis-isoxazole having monoterpenic skeleton, via a 1,3-dipolar cycloaddition reaction of (R)-Carvone-isoxazole with the nitrile oxide. The cycloaddition reaction was revealed to be highly regioselective. This regioselectivity was identified by a spectroscopic study of type HMBC and computational study (DFT). Cyclic voltammetry indicates that the oxidation potentials of compounds **2** and **3** vary depending upon the cycle count of the isoxazole. Moreover, DFT calculations were used to explain the regioselectivity for the formation of the bis-isoxazole, and the results are in good agreement with experimental findings.

### Spectral data for Bis-isoxazole 3

NMR Spectroscopy (500 MHz, CDCl3)

1H NMR spectrum

13C Decoupled 1H NMR spectrum

HRMS spectrum

HRMS spectrum

IR spectrum

IR spectrum

UV spectrum

## Figures and Tables

**Table 1:** The quantity of the volume of NaOCl added to the number of equivalents of dipole.

Number of equivalents	0.5	1	1.5	2	2.5	3	3.5	4
Volume of NaOCl (mL)	10	20	30	40	50	60	70	80
Yield obtained (%)	11	21	28	34	48	43	41	11

**Table 2:** IUPAC name, molecular structure, molecular formula, melting point, and analytical data of the studied isoxazole derivatives.

	IUPAC names	Structures	Analytical data
**2**	(R)-2-methyl-5-(5-methyl-3-(p-tolyl)-4,5-dihydroisoxazol-5-yl) cyclohex-2-en-1-one	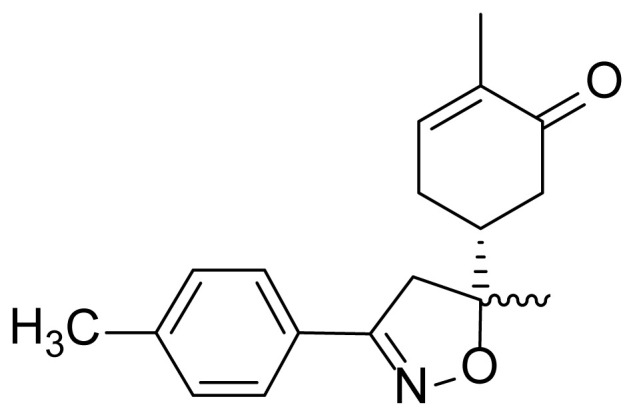	Chemical Formula: C_18_H_21_NO_2_ Exact mass: 306.1470 Molecular weight: 306.1466 Solid m.p: 96–98 °C
**3**	(3aS,5R,7aR)-7a-methyl-5-(5-methyl-3-(p-tolyl)-4,5-dihydroisoxazol-5-yl)-3-(p-tolyl)-3a,5,6,7a-tetrahydrobenzo[d] isoxazol-7(4H)-one	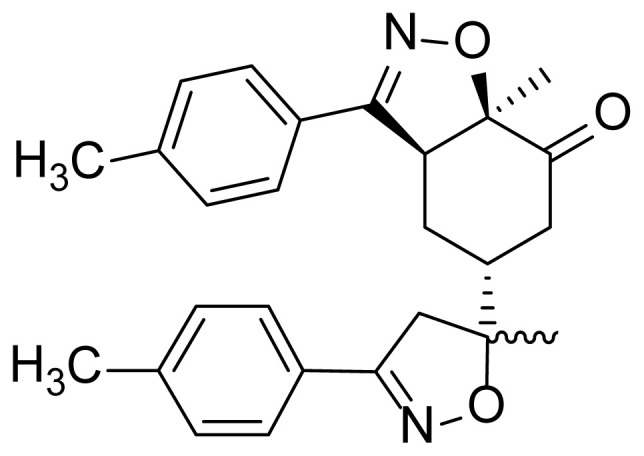	Chemical Formula: C_26_H_28_N_2_O_3_ Exact mass: 417.2127 Molecular weight: 417.2128 Solid m.p: 112–114 °C

**Table 3:** Effect of the 1,3-dipole amounts on the reaction yield.

**Quantity of dipole (eq)**	1	1.5	2	2.5	3	3.5	4
**Yield (%)**	11	21	28	34	48	43	39

**Table 4:** Experimental and theoretical (^1^H and ^13^C) NMR chemical shift (ppm) of studied diastereoisomers (**A1** & **A2**).

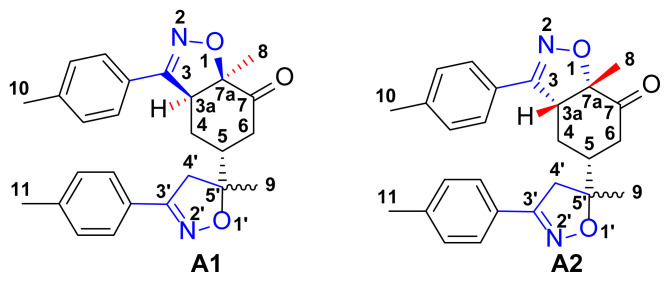							
^1^H NMR chemical shifts (ppm)				^13^C NMR chemical shifts (ppm)			
Atoms	Experimental (CDCl_3_)	Calculated DFT/B3LYP (ppm)		Atoms	Experimental (CDCl_3_)	Calculated DFT/B3LYP (ppm)	
		A1	A2			A1	A2
H-4′a	2.87 3.71 2.25 1.35 3.77 1.42 2.15 2.17 1.89 2.83	2.57	1.32	C4′	44.69 87.69 87.79 21.43 21.49 20.06 24.37 55.60 155.86 158.66 205.83	41.33	39.02
H-4′b		3.02	2.82	C5′		94.51	95.26
H-5		2.45	2.46	C7a		92.30	96.33
H-9		0.94	0.23	C10		21.54	19.85
H-3a		3.59	3.41	C11		21.56	19.88
H-8		1.39	1.61	C8		19.52	17.61
H-10		2.50	1.21	C9		25.27	23.01
H-11		2.51	1.16	C3a		59.71	61.66
H-6a H-6b		1.92 2.94	1.95 1.32	C3′ C3 C7		160.94 165.13 214.64	153.80 171.82 219.97

**Table 5:** Comparison of the experimental and theoretical vibrational spectra analysis of the compound. 3.

Assignment	Experimental FT-IR (cm^−1^) with KBr	Calculated (cm^−1^ ) B3LYP/6-311G(d,p)	
		A1	A2
ʋ(C-H) ʋ(C=O)_Ketone_ ʋ(C=N) ʋ(C=C) ʋ(CH2) ʋ(C-C) ʋ(N-O)	3439.32 1720.75 1615.09 1515.09 1444.60 1358.07 913.94	3113.07	3113.34
		1774.55	1777.19
		1655.39	1656.30
		1547.79	1547.69
		1495.13	1492.94
		1375.87	1375.32
		949.21	951.27

**Table 6:** Electronics properties and global reactivity indexes of compound **2** and dipole.

	μ(eV)	η (eV)	ω (eV)	N (eV)
**2**	−3.88	4.62	1.63	2.93
**Dipole**	−3.94	5.05	1.54	2.65

**Table 7:** Cyclic voltammetry parameters for the two molecules tested in a 0.1 M Et_4_NClO_4_ /acetonitrile solution at room temperature.

	Molecules	2	3
**Oxidation Curves (I)**	E_ox_^onset^	−1.424	−1.639
	E_red_^onset^	-	-
	E_HOMO_	−3.376	−3.161
	E_g_^CV^ (ev)	−1.424	−1.639
	ΔEp (mV)^(a)^	1.224	1.463
**Reduction Curves (II)**	E_ox_^onset^	-	-
	E_red_^onset^	−0.688	−0.808
	E_LUMO_	−4.112	−3.992
	E_g_^CV^ (ev)	0.688	3.992
	ΔEp (mV)^(a)^	1.031	1.255

(a) $\Delta {\text{E}}_{\text{p}}=\left|{\text{E}}_{\text{p}}^{\text{c}}-{\text{E}}_{\text{p}}^{\text{a}}\right|$

**Table 8:** Absorption edge and band gap energy of compounds **2** and **3**.

Compound	Assignments		Absorption edge (nm)	Band gap energy (E_g_^Opt^ (eV))

**2**	π	π^*^	217.12	5.72
	n	π^*^	232.09	5.35

**3**	π	π^*^	217.12	5.72
	n	π^*^	231.30	5.37
