# Supplementary figures and images for: New bis-isoxazole with monoterpenic skeleton: regioselective synthesis, spectroscopic investigation, electrochemical, and density functional theory (DFT) studies
Source: Turk J Chem. 2021 Dec 18;46(2):506–22. doi: 10.3906/kim-2109-28 (PMC10734740; doi:10.3906/kim-2109-28)

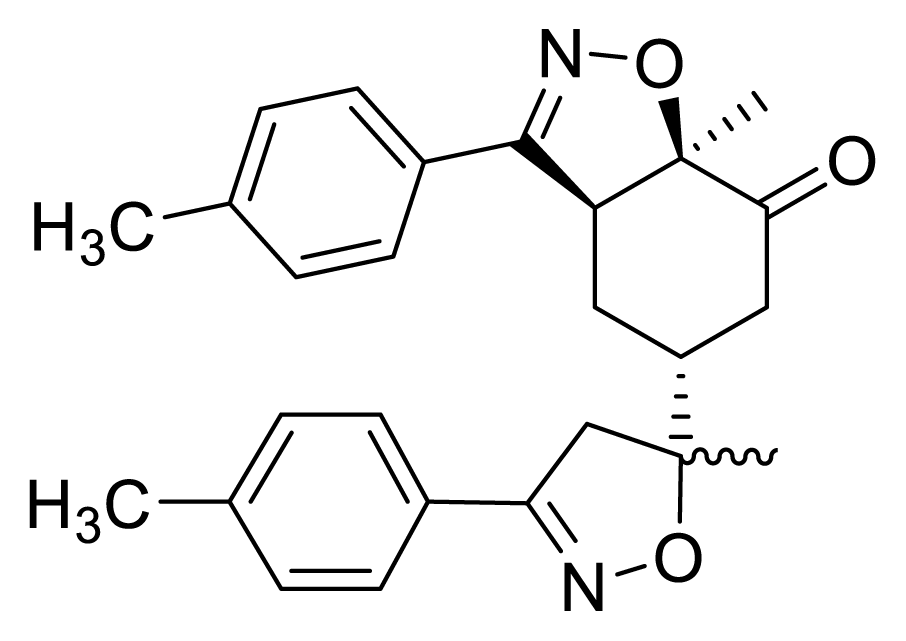

Supplement: Supplementary file 1 — NMR Spectroscopy (500 MHz, CDCl3) [file turkjchem-46-2-506s1.tif]

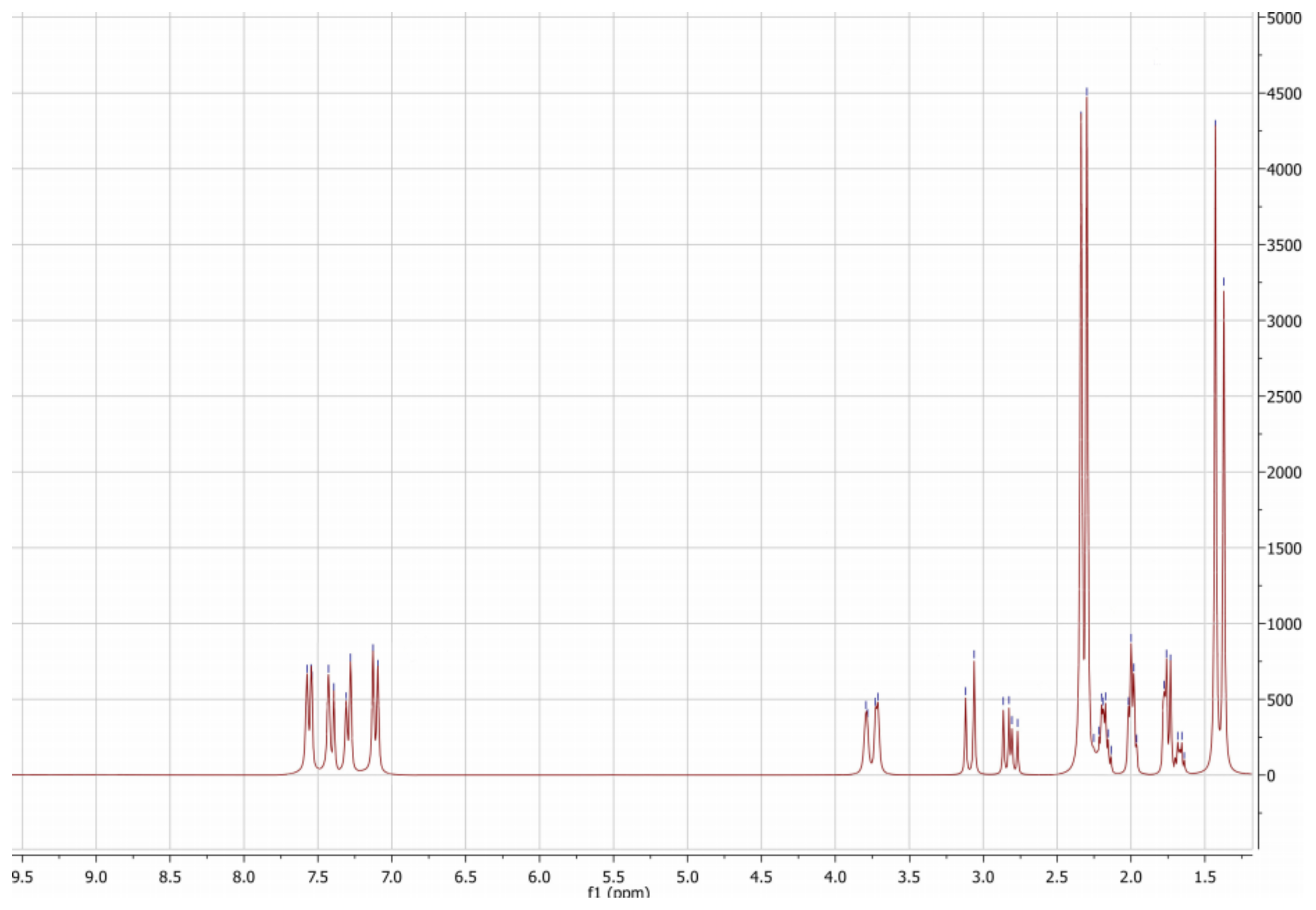

Supplement: Supplementary file 2 — 1H NMR spectrum [file turkjchem-46-2-506s2.tif]

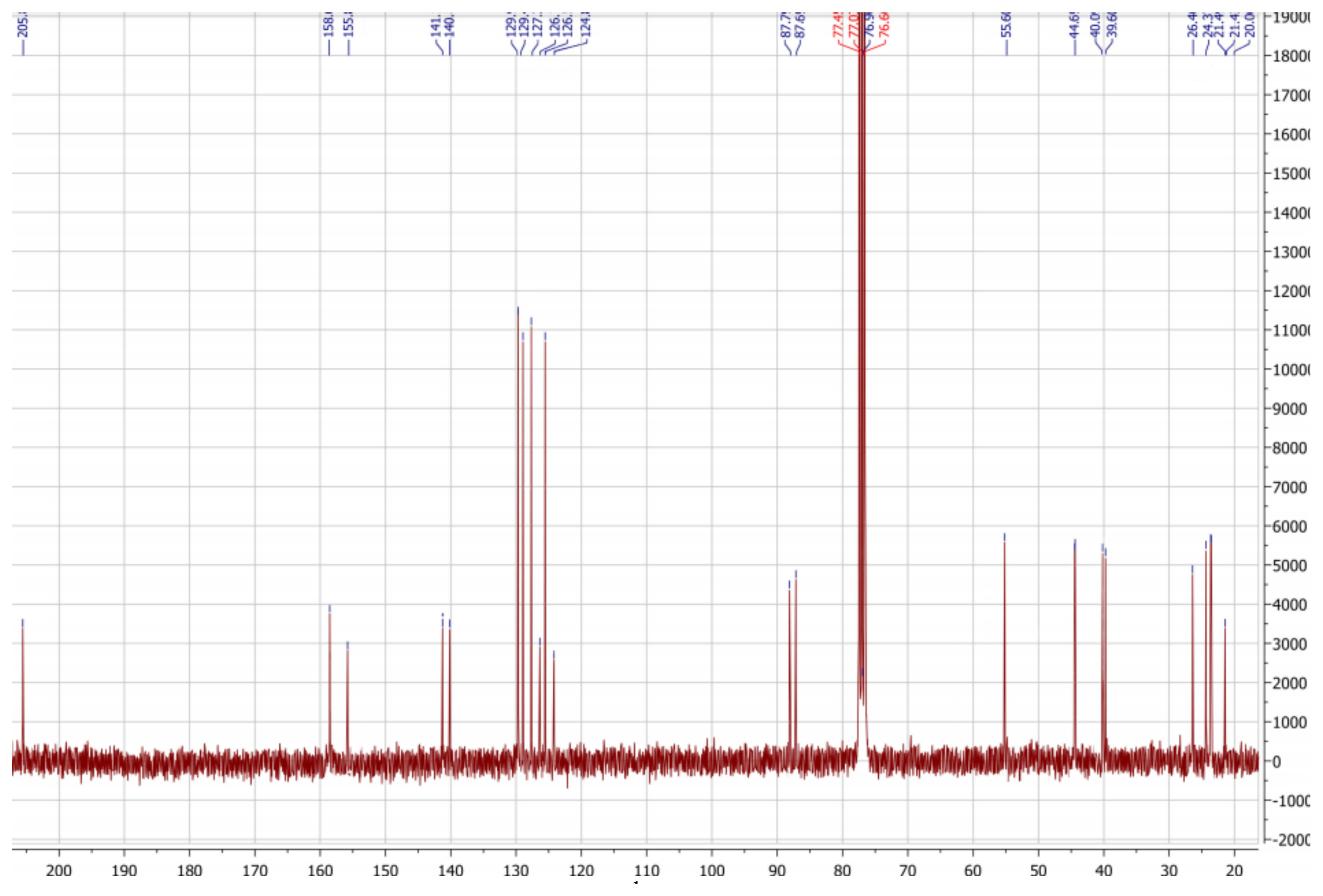

Supplement: Supplementary file 3 — 13C Decoupled 1H NMR spectrum [file turkjchem-46-2-506s3.tif]

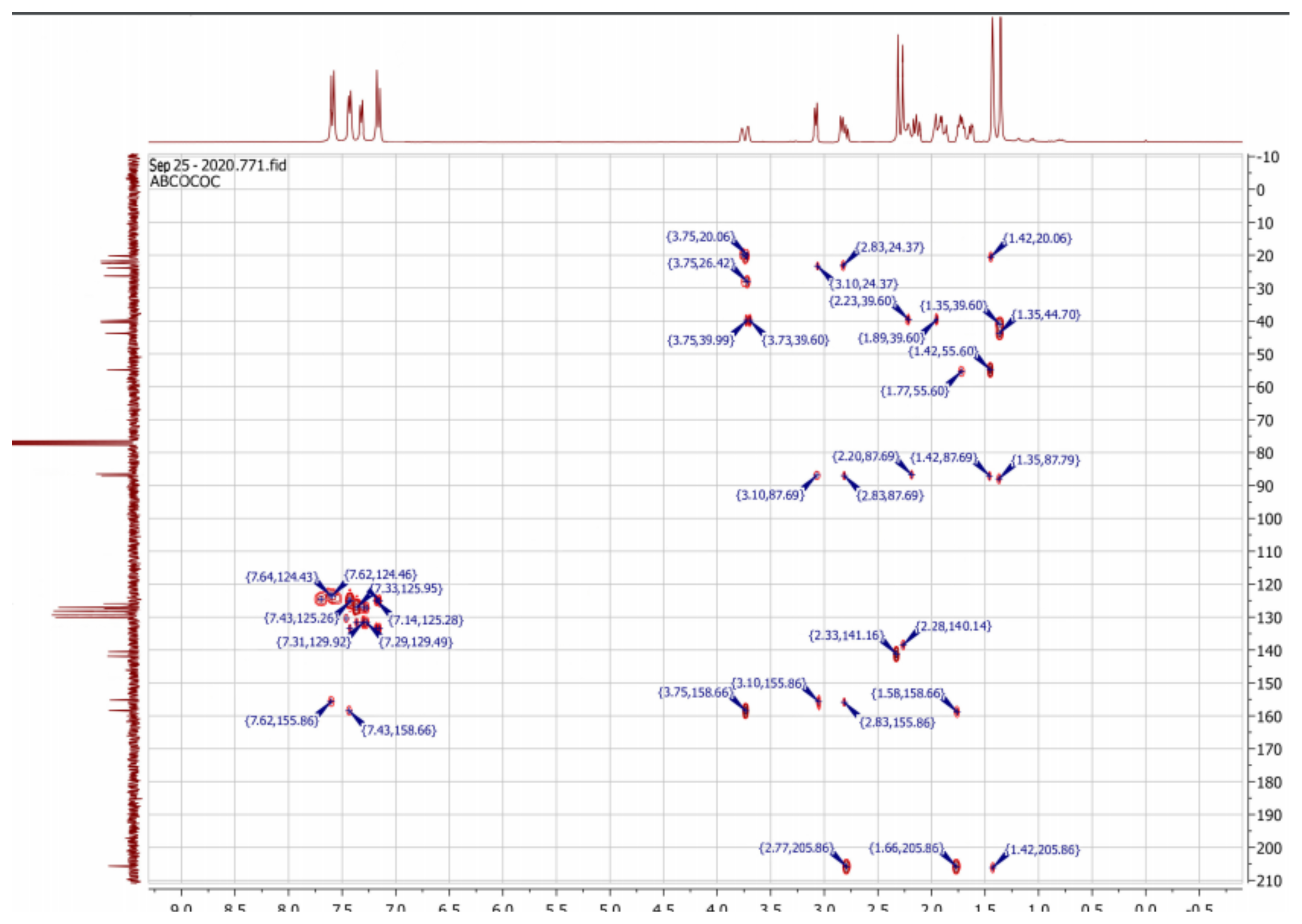

Supplement: Supplementary file 4 — HRMS spectrum [file turkjchem-46-2-506s4.tif]

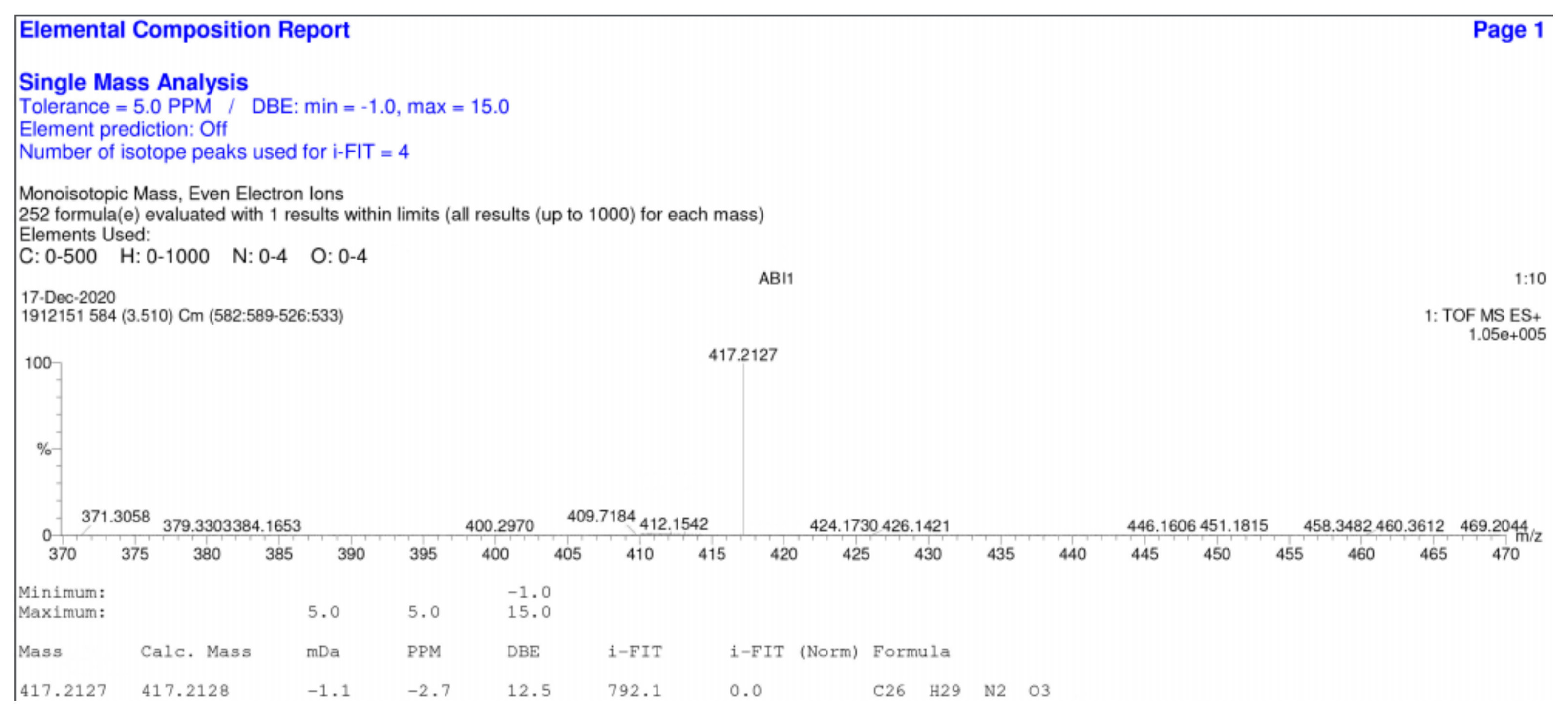

Supplement: Supplementary file 5 — HRMS spectrum [file turkjchem-46-2-506s5.tif]

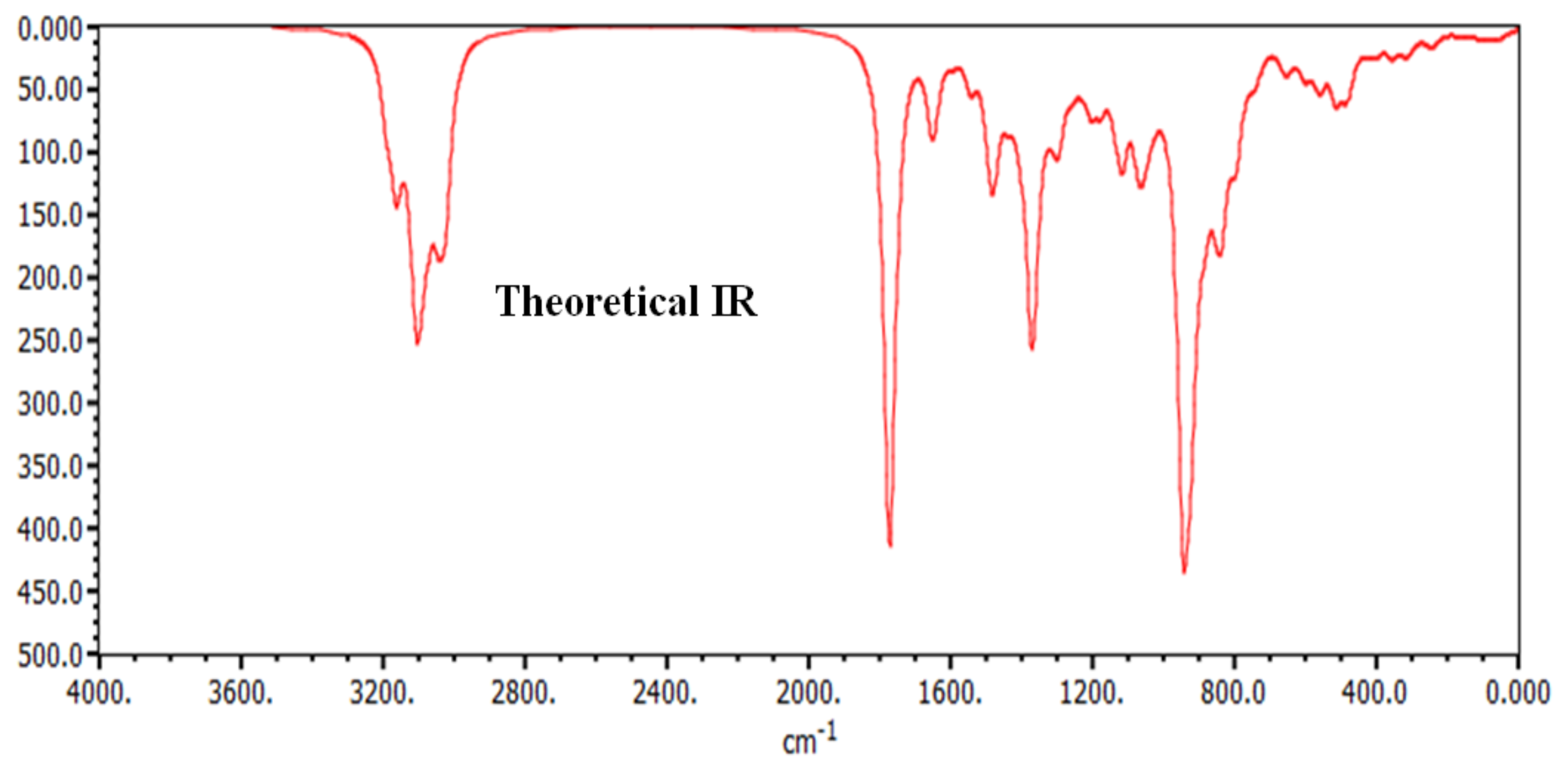

Supplement: Supplementary file 6 — IR spectrum [file turkjchem-46-2-506s6.tif]

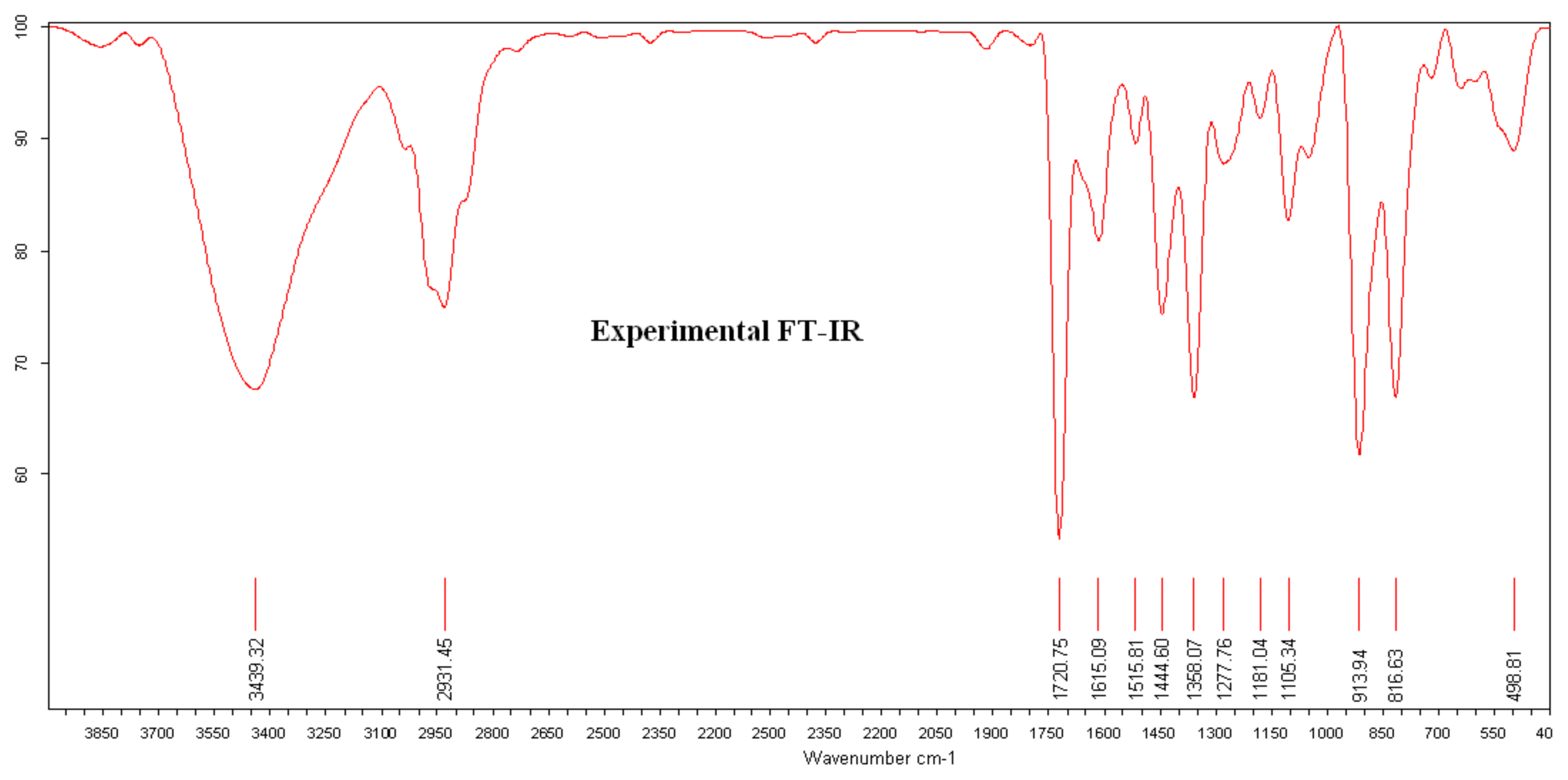

Supplement: Supplementary file 7 — IR spectrum [file turkjchem-46-2-506s7.tif]

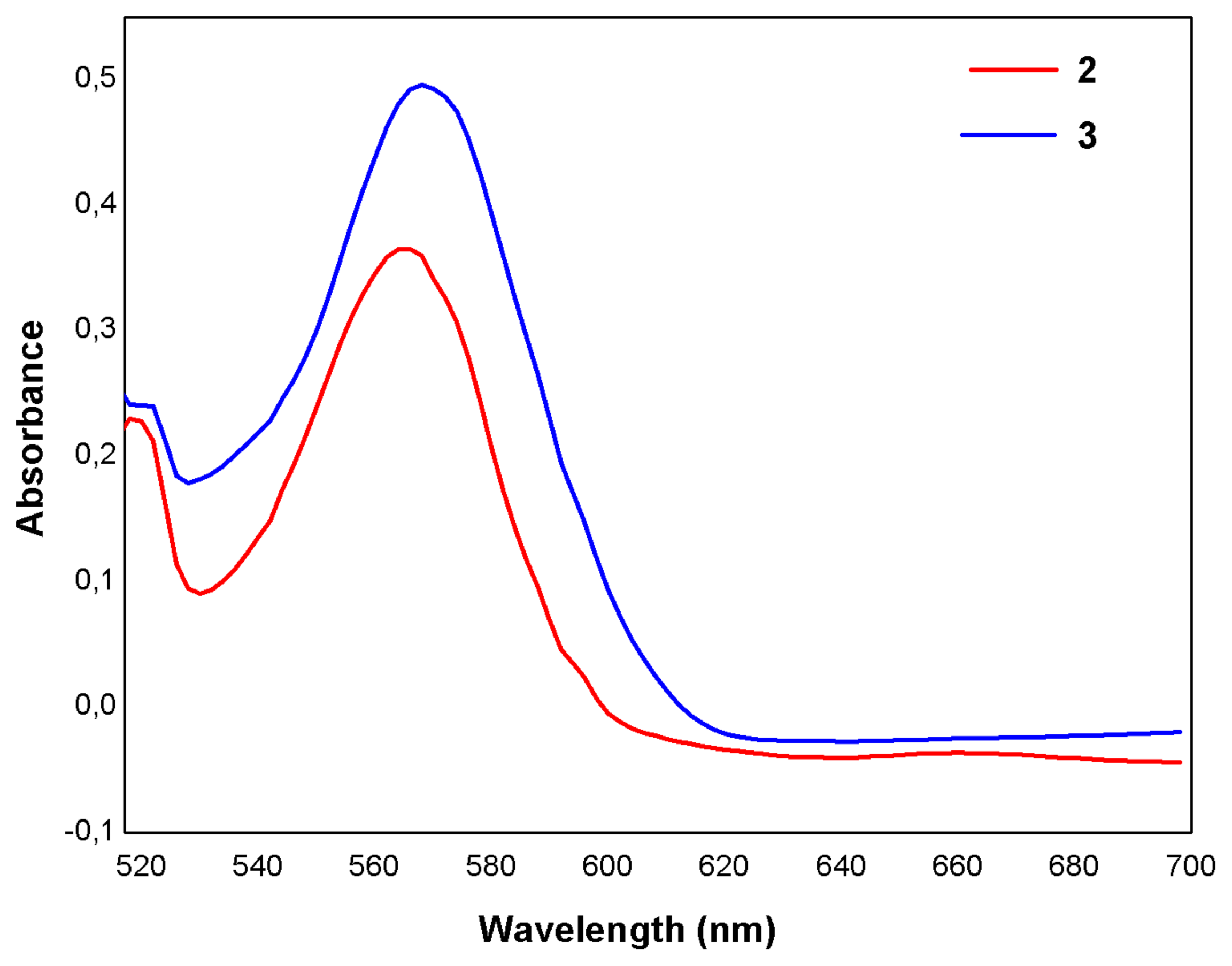

Supplement: Supplementary file 8 — UV spectrum [file turkjchem-46-2-506s8.tif]
